# Unsupervised self-organising map classification of Raman spectra from prostate cell lines uncovers substratified prostate cancer disease states

**DOI:** 10.1038/s41598-024-83708-6

**Published:** 2025-01-04

**Authors:** Daniel West, Susan Stepney, Y. Hancock

**Affiliations:** 1 Department of Computer Science, University of York, Heslington, York, YO10 5DD UK; 2 School of Physics, Engineering and Technology, University of York, Heslington, York, YO10 5DD UK; 3 York Biomedical Research Institute, University of York, Heslington, York, YO10 5DD UK

**Keywords:** Biophysics, Cancer, Cell biology, Optics and photonics, Optical techniques, Optical spectroscopy, Raman spectroscopy

## Abstract

Prostate cancer is a disease which poses an interesting clinical question: Should it be treated? Only a small subset of prostate cancers are aggressive and require removal and treatment to prevent metastatic spread. However, conventional diagnostics remain challenged to risk-stratify such patients; hence, new methods of approach to biomolecularly sub-classify the disease are needed. Here we use an unsupervised self-organising map approach to analyse live-cell Raman spectroscopy data obtained from prostate cell-lines; our aim is to exemplify this method to sub-stratify, at the single-cell-level, the cancer disease state using high-dimensional datasets with minimal preprocessing. The results demonstrate a new sub-clustering of the prostate cancer cell-line into two groups—protein-rich and lipid-rich sub-cellular components—which we believe to be mechanistically linked. This finding shows the potential for unsupervised machine learning to discover distinct disease-state features for more accurate characterisation of highly heterogeneous prostate cancer. Applications may lead to more targeted diagnoses, prognoses and clinical treatment decisions via molecularly-informed stratification that would benefit patients. A method that could discover distinct disease-state features that are mechanistically linked could also assist in the development of more effective broad-spectrum treatments that simultaneously target linked disease-state processes.

## Introduction

Cancer is one of the leading causes of death worldwide, with prostate cancer being the second-leading cause of cancer deaths in males^1^. The incidence of prostate cancer is high with one-in-eight males being diagnosed during their lifetime and the number of new cases set to double in the next twenty years from 1.4M in 2020^1,2^. Prostate cancer is a challenging disease of complex molecular structure and high heterogeneity across all length scales, both within a single patient and between patients^3^. Its molecular heterogeneity is difficult to assess and quantify in solid tumours, thereby limiting an individual’s classification of risk for aggressive disease and their targeted treatment options^4^. This work looks at prostate cancer as a model disease, both because it is common and because it poses an unusual clinical question amongst cancers: Should it be treated? Many forms of prostate cancer are relatively indolent, and only a small subset are highly malignant and aggressive^5^. Benign growth of normal prostate tissue can also cause similar symptoms (e.g.,^6^). The clinical pathway to diagnosis is therefore complex and lengthy. Given the large variation in how the disease presents and progresses, conventional methods involving imaging and histology are limited in capturing the full breath of disease heterogeneity. Multiparametric MRI has good negative predictive outcome (> 90%)^7^, but is poor at detecting clinically significant disease (positive predictive rate of approx. 35%)^8^. Histopathology provides optical classification of tumour grade (Gleason scale), but is limited by interpretation variability and inability to discern certain forms of prostate cancer (e.g., cribriform and and intraductal carcinoma of the prostate)^9,10^. Both methods remain restricted at their points of resolution. Significant variability also exists in clinical pathways and disease stability. For example, only 15% of patients require immediate treatment intervention^11^, yet 27% of those deemed “indolent” progress within five years post diagnosis^12^. An urgent question to address from the molecular scale is why?

The significance of molecular heterogeneity in understanding prostate-cancer disease and its translation to precision diagnostics, risk stratification and more effective personalised treatments cannot be underestimated^13^. Recent evidence shows heterogeneity in tumour-patterns sub-stratified in histopathology with distinct prognostic outcomes beyond the Gleason score^14–16^. Woodcock et al. have also determined multiple “evo” subtypes of prostate cancer by classifying its genomic evolution^17^. Therefore, having a tractable means of quantifying molecular heterogeneity to the sub-cellular length scale is essential. To assist with molecular disease characterisation and heterogeneity assessment, spectroscopic methods, such as Raman spectroscopy, can also be used for non-destructive and label-free testing of cells, and the environmental impact on cellular behaviour^18^. Raman spectroscopy is a physical process that involves inelastic photon-molecule scattering for “fingerprinting” at the molecular length scale^19^. Its advantage is in holistic sampling of biological systems with capture of complete and intact cellular information. Applied to cellular studies of prostate cancer, Raman spectroscopy has uncovered specific protein, lipid and DNA/RNA changes that stratify malignant-state characteristics and has revealed mechanistic differences of the disease state^20–25^.

In this paper, we apply unsupervised machine learning using self-organising maps (SOMs) to assess the capability for molecular stratification of live-cell Raman spectroscopy data acquired from a metastatic prostate cancer cell line (LNCaP)^26,27^ and normal prostate cell line (PNT2-C2)^28–30^. Within the context of an unsupervised classification scheme, the datasets are unlabelled, thereby allowing the method to determine its own set of classification rules. Kohonen introduced SOMs in the 1980s as a new means of classification that use topological network organisation to map high-dimensional data onto a two-dimensional array^31,32^. Hence, SOMs provide a convenient visualisation of cluster stratification that is not otherwise apparent. The application of SOMs to Raman spectroscopy and unsupervised machine-learning, in general, is still in its infancy with most research focused on supervised classification methods, such as PCA-LDA^33^. To the best of our knowledge, there are only a few publications that have applied SOMs to biological-systems research, namely, to animal tissue^34,35^ and human cell lines^36,37^. In cell-line studies, Harris et al.^36^ analysed Raman spectra from thyroid cancer and normal thyroid cells with >90% accuracy in SOM classification of the disease state. Majumdar et al. used a SOM to distinguish Raman results of the differentiation stages of a monocytic (macrophage) cell line^37^. Both studies applied supervised learning and, as such, did not explore the full capability of the method.

Of particular interest in this work is the unsupervised learning within SOMs, which allows for the discovery of molecular stratification. In testing the SOM for this purpose, the choice of Raman data from LNCaP (cancer) and PNT2-C2 (normal) prostate cell lines provides an important challenge for sub-classification, as standard cell lines are considered inherently homogeneous^38^. To benchmark the SOM’s capability, we have also compared the results to standard PCA-LDA classification. Further, we test the SOM on the region of Raman spectra from 2700 to 3600 cm^−1^, the so-called high-wavenumber region, which in prostate cells encapsulates important mechanistic information pertaining to the cancer state^23,24^. This region informs about the unsaturated-to-total fatty-acid ratio, cholesterol/cholesterol-ester use, and lipid-droplet formation; all key factors in how prostate cancer reroutes its energy formation and uses it to its advantage^39,40^. The use of Raman spectroscopy within this context could also provide an easy and complimentary means to acquire state-level discernment of molecular heterogeneity in cancer at the single-cell level. In the field of cancer research, such a tractable means of determining molecular sub-classification could lead to the discovery of new stratified disease states, and hence, more targeted and risk-stratified treatment decisions could ensue. The visual SOM also has benefit for clinicians: It is a readily interpretable and understandable illustration by which patients could see the position of their own cellular signatures within the complex domain of benign and malignant patterns.

## Results

### Univariate analyses, multivariate PCA, and PCA-LDA classify the high-wavenumber component of the LNCaP disease state

**Fig. 1:**
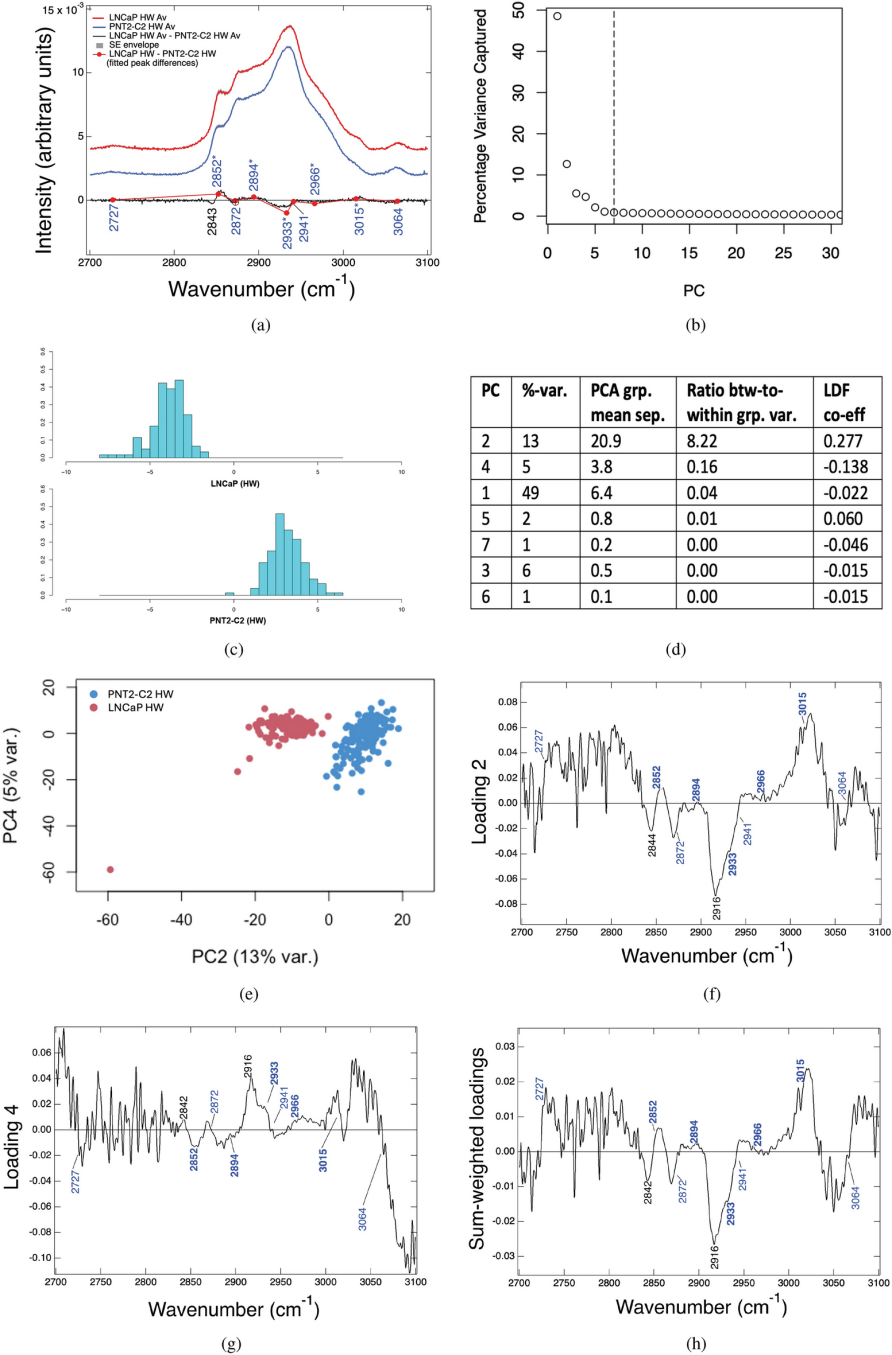
(**a**) Spectral averages plus SE envelopes for LNCaP and PNT2-C2. (Lower) Spectral-subtraction plot (black), and Gaussian peak-fitted intensity differences plus propagated SE uncertainties (red). (**b**) Percentage variance captured versus PC number. The vertical line shows the maximum 7 PCs used in the LDA (total ~ 77% variance captured). (**c**) LDA histogram (classification result). (**d**) Table showing the PC number, percentage variance captured, group-mean separations, ratio of the between- to within-group variances and LDF coefficients. (**e**) PCA scatterplot for high-wavenumber LNCaP versus PNT2-C2 (PC2$\times$PC4). Loadings for (**f**) PC2, (**g**) PC4 and the (**e**) sum-weighted loadings. Key wavenumber positions are shown.

The average, high-wavenumber fingerprints of the PNT2-C2 (normal-prostate) and LNCaP (metastatic prostate-cancer) cell lines obtained using live-cell Raman spectroscopy are shown in Fig. 1a. The total dataset comprises 154 single-cell point spectra for PNT2-C2 and 130 single-cell point spectra for LNCaP, with each spectrum containing *N* = 1056 wavenumber points. The spectral datasets are statistically converged (SI Section A), thereby ensuring that the converged spectral averages faithfully represent the population-level sampling. The molecular heterogeneity across these cell lines is represented by the converged standard-error (SE) envelopes, which are displayed over the spectral averages (Fig. 1a). A spectral-difference plot (black line) was also obtained by subtracting the average spectrum of PNT2-C2 (normal) from the LNCaP (cancer) cell line thereby defining the relative disease state.

Gaussian peak-fitting to the average spectra was performed to determine the de-convolved peak positions and peak-intensity differences resolved to these bands (Fig. 1a). Table 1 shows the quantitative values associated with the peak-intensity differences together with the fitted standard-error uncertainties and peak assignments. The up-regulation ($\uparrow$) and down-regulation ($\downarrow$) of the LNCaP cancer state relative to the PNT2-C2 normal baseline (column 4, Table 1) shows favourable comparison with results from the literature^23,24^. Key differences from these results are found at 2852, 2894, 2933, 2966 and 3015 cm^−1^ (Table 1). A statistical comparison between LNCaP and PNT2-C2 was also performed using PCA, where PC1 captures the maximum proportion of the total variance at 49%, followed by PC2 at 13%, PC3 at 6%, and PC4 at 5% total-variance weightings. The plot of the percentage variance per PC shows PC7 at 0.9% of the total variance captured to be just past the elbow of the plot (Fig. 1b). This result confirms 7 PCs to be a viable minimum number for the PCA-LDA classification (i.e., reduced dimension) versus the 1056 initial wavenumber variables per spectrum. Within LDA classification, the linear-discriminant function (LDF) separates group clusters in PCA space by maximising the between-group and minimising the within-group variances relative to the cluster centroids, such that LDF = $w_1$PC1 + $w_2$PC2 ... + $w_n$PC*n*, where $n=7$ in this specific example, and $w_i$ are the corresponding LDF coefficients, also referred to as weights. Graphical representation of this classification result with leave-one-out cross validation (LOOV) (Fig. 1c) shows separation of the PNT2-C2 and LNCaP groups via stacked histograms that encapsulate the action of the LDF on the group-separated PCA data. A stable 99.6% average accuracy was determined over 1000 iterations in the LOOV-classification with the maximum number of 7 PCs included.

**Table 1:** Peak-intensity differences between Gaussian peak-fitted bands in LNCaP (cancer C) relative to PNT2-C2 (normal N baseline) taken from the averaged spectral fingerprints in the high-wavenumber region.

Band (cm^−1^)	Proposed peak assignments for whole-cell, nucleus sampling	$\delta$ [C−N] ± SE [E-4]	C$\uparrow$ or $\downarrow$
2727	Lipids: C−H $\hbox {stretches}^{\dag }$	0.43 ± 0.08	C$\uparrow$
2852*	Lipids: $\hbox {CH}_2$ $^{\dag }$	**5.1 ± 0.5**	C$\uparrow$ $^{a\uparrow , c\uparrow }$
2872	Lipids and Proteins: $\hbox {CH}_2$ & $\hbox {CH}_3$ $^{\dag }$	− 0.020 ± 0.001	C$\downarrow$ $^{a\uparrow , b\downarrow }$
2894*	Lipids and Proteins: CH, $\hbox {CH}_2$ & $\hbox {CH}_3$ $^{\dag }$	**2.83 ± 0.04**	C$\uparrow$ $^{a\uparrow }$
2933*	Proteins and Lipids: $\hbox {CH}_2$ & $\hbox {CH}_3$ $^{\dag }$	− **9.7 ± 0.2**	C$\downarrow$ $^{b\downarrow }$
2941	Proteins and Lipids: C−H lipids & proteins; $\hbox {CH}_2$ modes in $\hbox {lipids}^{\dag }$	− 0.75 ± 0.08	C$\downarrow$ $^{a\downarrow , b\downarrow }$
2966*	Lipids: $\hbox {CH}_3$ asymmetric stretch; cholesterol & cholesterol $\hbox {ester}^{\dag }$	− **2.41 ± 0.06**	C$\downarrow$ $^{a\downarrow , b\downarrow }$
3015*	Lipids: unsaturated =CH stretch in $\hbox {lipids}^{\dag }$	**1.39 ± 0.01**	C$\uparrow$ $^{a\uparrow , b\downarrow }$
3064	Proteins: C−H stretch; phenylalanine, tyrosine & $\hbox {tryptophan}^{\ddag }$	− 0.54 ± 0.02	C$\downarrow$

Column 1: Average peak positions. Column 2: Proposed peak assignments showing those bands, which are predominant in lipids (when listed first) and proteins (when listed first). Column 3: Measured peak-intensity difference ($\delta$) in cancer minus normal [C−N] ± the propagated SE uncertainty. Column 4: Cancer (C) up-regulated ($\uparrow$) or down-regulated ($\downarrow$) against the normal baseline (this work). The superscripts denote the corresponding literature comparisons (see also the table footnotes below) with $\uparrow$ and $\downarrow$ indicating the comparative up-regulation and down-regulation of cancer in those works, respectively. Significant values are highlighted in bold.

$^a$LNCaP and PC3 metastatic cells lines. Untreated versus treated. Formaldehyde fixed^23^. LNCaP comparison shown. Lipid-droplet sampling.

$^b$PC3 and LNCaP versus PNT2 normal prostate cell line. Cells fixed with paraformaldehyde^24^. LNCaP comparison shown. Whole-cell sampling.

$^c$PC3 and LNCaP versus PNT2 normal prostate cell line. Cells fixed with paraformaldehyde^24^. LNCaP comparison shown. Liquid-droplet sampling.

Peak-assignment references also include reference ^19^$$^\dag$$ and reference ^25^$$^\ddag$$

Inspection of the PCA-LDA result shows PC2 and PC4 to have the highest LDF weights in magnitude (Fig. 1d, column 4) and hence the greatest effect on the LDA classification (Fig. 1c). Indicatively, the PCA result for PC2 and PC4 (Fig. 1e) shows LNCaP and PNT2-C2 to have very good separation along the PC2$\times$PC4 axis, albeit with some outliers, which we will later address within the context of the SOM. PCA loadings corresponding to the PC2 and PC4 reduced dimensions (Fig. 1f,g), and a sum LDF-weighted loadings taken over all seven PCs are also shown (Fig. 1h). Quantitative analysis of the PCA result within the context of the PCA-LDA loadings demonstrates features that are commensurate with the spectral subtraction findings (cf. Fig. 1a,h).

The LNCaP cell line shows marked changes in the lipids as evidenced by C$\uparrow$ for the predominant lipid markers in Table 1. The $\hbox {CH}_2$ lipid band at 2852 cm^−1^ assigned to total fatty acids is increased in LNCaP relative to PNT2-C2 ($\delta =5.1$C$\uparrow$). This result indicates that in the live-cell result there is a greater amount of lipids in LNCaP (cancer) relative to PNT2-C2 (normal). The relative increase in the 3015 cm^−1^ marker ($\delta$ = 1.39C$\uparrow$) also shows an increase in unsaturated lipid content, and lipids in general, in live-cell LNCaP (2894 cm^−1^ increase). The exception is the reduction in the cholesterol and cholesterol-ester (CE) band at 2966 cm^−1^ in the LNCaP cancer state relative to PNT2-C2, which we interpret as indicating cholesterol synthesis from cholesterol-ester and cholesterol use, i.e., that the LNCaP cells have less requirement for cholesterol-ester storage^41^. The result also correlates with a key peak-intensity ratio marker, 3015/2966, which we measured as 0.100 ± 0.002 for LNCaP versus 0.065 ± 0.001 for PNT2-C2, with the relative up-regulation of this marker in LNCaP cancer (C$\uparrow$) also being indicated in Hislop et al.^24^. Our findings (Table 1) show the live-cell results to have features in keeping with lipid-droplet analyses in other works, e.g., Refs.^23,24^. We conclude this finding to be a reasonable outcome within the statistics of our measurements as LNCaP cells have been measured to have more liquid droplets on average than PNT2-C2^24^. In comparison, key protein-dominant markers (in particular at 2966 cm^−1^) are shown to be down regulated in LNCaP cancer (C$\downarrow$) indicating increased protein metabolism in AR-positive LNCaP. This finding is also supported in the literature. For example, Ahmad et al.^42^ states “enhanced” protein uptake and metabolism in prostate cancer as key features correlated to AR-signalling.

**Fig. 2:**
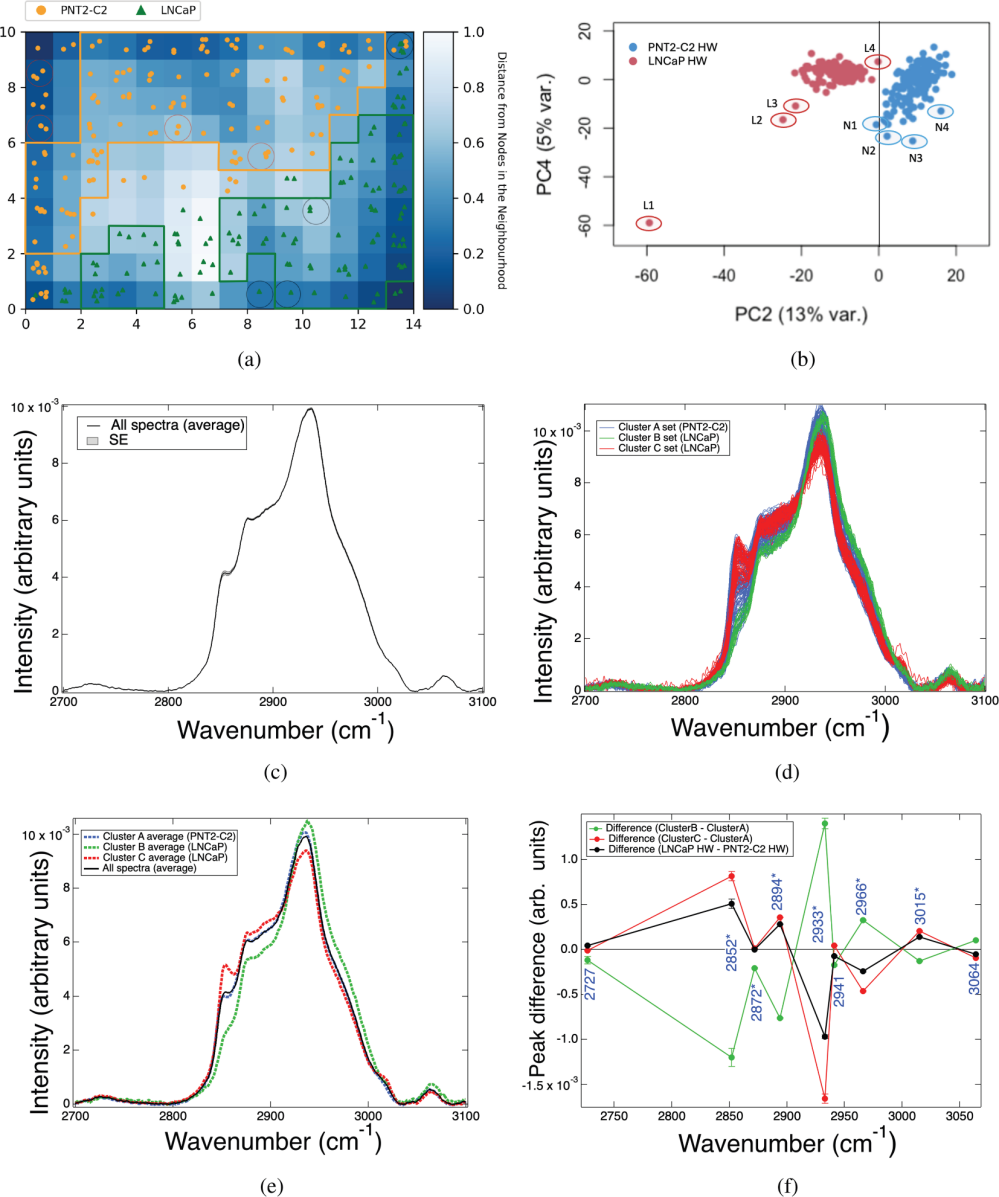
(**a**) Unsupervised SOM map of dimension 14$\times$10 from the total data set of LNCaP and PNT2-C2 spectra (originally blinded), with nodes (0,0) bottom left to (13,9) top right. Observations per node are shown with selected PCA points of interest (**b**) circled. Unsupervised classification of PNT2-C2 (Cluster A, upper-left; yellow bordered) and the discovery of two sub-stratified LNCaP clusters (B: lower left, and C: lower right; green bordered) are indicated. Due to open-boundaries, corner nodes are excluded as are edge nodes with nearest-neighbours that have no observations. L2 (8,0), L4 (13,9), N1 (0,6) and N3 (0,8) meet these criteria. L4 and N1 also cross PC2 = 0 with PC2 being highly-weighted in the loadings. L3 (9,0) is included as (9,1) is populated (~ 1% Cluster C). Nodes with a distance score (DS) $\le$ 0.72 (threshold distance) are included, e.g., N2 (5,6) and N4 (8,5) (~ 2% Cluster A). L1 (10,3) is a PCA outlier with borderline inclusion at DS = 0.71 (~ 1% Cluster C). Cf. Methods section, Table 2 & SI Section C. (**c**) Average spectrum for the combined prostate cell-line dataset (LNCaP + PNT2-C2) with SE envelope. (**d**) Spectral distributions for each SOM-identified cluster. Cluster A (PNT2-C2) has a broad distribution. Clusters B and C (both LNCaP) have well-separated, distinct distributions. (**e**) Cluster separated, average spectra for SOM-identified Clusters A, B and C compared to the average spectrum for the full dataset. (**f**) Gaussian-fitted, peak-intensity differences from the average spectral profiles relative to the normal-baseline results, with Cluster B (sub-stratified as protein dominant) relative to Cluster A, and Cluster C (sub-stratified as lipid dominant) relative to Cluster A, versus the full dataset for LNCaP relative to PNT2-C2. These relative differences define the disease-state biomarkers in the sub-stratified B and C clusters, and in the full dataset result (see also Fig. 1a). Propagated SE uncertainties are shown.

### Unsupervised self-organising map (SOM) classification reveals sub-stratification of the LNCaP (cancer) disease state

An unsupervised SOM was used to assess the potential for sub-classification of the spectral results. SOMs (also known as Kohonen maps) are a visual representation of network-connected units (nodes) that assume the topology of an input dataset^31^. With each round of training, the best matching unit (BMU) is defined as the node that best maps to an input observation pattern (in this case, a vectorially-defined spectrum) using a relative-distance mapping process, i.e., via a *competition* that tests for the minimum relative distance to it. The BMU, in turn, then mathematically exerts an effect on neighbouring nodes to bring those that are relationally similar to it closer in relative distance, thereby uncovering clusters of patterns within the dataset. The process of competitive feedback when training with a dataset enables the nodes to recognise and establish unique relational patterns within the input data^32^. The algorithm for the SOM approach is described in the Methods section.

A rectangular map is used for the SOM as it is more easily interpretable. Figure 2a shows the classification result from the SOM determined from a randomised, blind dataset comprising both the LNCaP and PNT2-C2 spectral data. Here, the distance from the neighbourhood nodes (right colour scale on the heat map) refers to the converged relative distances normalised to a scale from 0 to 1. The SOM shows an unsupervised classification result of a single cluster for PNT2-C2 (Cluster A: upper-left triangle of the SOM map), and two sub-stratified clusters in LNCaP (Cluster B: lower left, and Cluster C: lower right of the SOM), separated by a maximum-distance separator (i.e., white nodes) at (7,2), (7,3) and (7,4). The mapping of each spectrum to the SOM is shown as a single data point, with sub-clusters identified from regional areas of nodes that meet a distance score (DS) of $\le$ 0.72 threshold distance, with this value corresponding to the minimum distance where clefts between clusters (i.e., cluster borders) occur. Due to open-boundary conditions, corner nodes are excluded in the SOM classification, as are edge nodes that have internal nearest-neighbour nodes with no observations. PCA points of interest due to their PC scores, hence distances from the PCA cluster centres, are highlighted in Fig. 2b and circled in the SOM map (Fig. 2a). L2 (8,0), L4 (13,9), N1 (0,6) and N3 (0,8) meet these criteria. L4 (corner node) and N3 (edge node) also cross PC2 = 0, with PC2 being the highest-weighted component in the loadings (cf. Fig. 1d). PCA L3 (9,0) is included in the SOM classification as its neighbouring internal node (9,1) is populated as per our inclusion criteria (L3 = ~ 1% weighting in Cluster C; cf. Table 2). PCA N2 (5,6) and N4 (8,5) are included with DS $\le$ 0.72 values (total ~ 2% weighting in Cluster A; cf. Table 2). PCA L1 (10,3) is a PCA outlier, which is borderline for inclusion at DS = 0.71 (L1 = ~ 1% weighting in Cluster C). Cf. also the SOM methods section and SI Section C. The discovery of the two sub-stratified clusters in LNCaP from the unsupervised SOM (Clusters B and C) is a new finding against the results from PCA. The low percentage weighting of possible PCA outliers in the cluster definitions confirm the unsupervised SOM classification to be statistically significant.

**Table 2:** Cluster distributions and their locations in the SOM (cf. Fig. 2a).

Cluster	Location	PNT2-C2	LNCaP	Subset (%)	Total (%)
A	Top half (mostly left)	121	0	79	43
B	Lower left	0	18	14	6
C	Lower right	0	73	56	26

Column 1: Cluster name. Column 2: Cluster location in the SOM. Columns 3 and 4, respectively: Number of PNT2-C2 and LNCaP observations per cluster. Column 5: Percentage (%) proportion of observations relative to their respective subset number of spectra (PNT2-C2 = 154; LNCaP = 130). Spectra mapped to SOM nodes that are excluded from the cluster definitions comprise the remaining percentage of spectra. Column 6: Percentage (%) proportion of observations relative to the total number of spectra in the combined dataset across all spectra (*n* = 284).

The full set of SOM-classified clusters and their percentage weightings within the full set of spectra (combined), and relative to the separate LNCaP and PNT2-C2 datasets, are shown in Table 2. The spectral average across the full set of spectra (LNCaP and PNT2-C2 combined) (Fig. 2c), relative distributions of the SOM-classified spectra in each cluster (A, B and C) (Fig. 2d), and the spectral average per cluster (Fig. 2e) can also be compared. Although Cluster A (79% PNT2-C2 spectra) exhibits a broad distribution, the LNCaP sub-stratified clusters B and C (total 70% of the LNCaP spectra) are found to be statistically distinct and well separated (Fig. 2d). The high percentage weighting of spectra that have been classified indicate these results to be statistically significant. In this respect, the SOM faithfully finds through unsupervised classification, the naturally contained subclasses of complex patterns within the combined Raman data set. To determine the biomolecular differences between the SOM clusters, the average spectra in Fig. 2e were Gaussian peak-fitted, with the results of the disease-state clusters (B and C) defined relative to the normal-state cluster (A). Figure 2f shows the peak-intensity differences for the sub-stratified clusters B and C relative to A, with these results also compared to the peak-intensity profile for the LNCaP minus PNT2-C2 disease state obtained using the full set of spectra (Table 1 and Fig. 1a). Key differences occur at 2852 ($\hbox {CH}_2$ total fatty-acid), 2872 ($\hbox {CH}_2$ and $\hbox {CH}_3$ lipids and proteins), 2894 (CH, $\hbox {CH}_2$, & $\hbox {CH}_3$ primarily lipids), 2933 ($\hbox {CH}_3$ primarily proteins), 2966 ($\hbox {CH}_3$, cholesterol and cholesterol ester), and 3015 cm^−1^ ($=$CH unsaturated lipids). Although the peak-intensity difference plot for Cluster C in the disease state follows the same trend as the full-population study, Cluster B is shown to be opposite in trend.

**Table 3:** Key, peak-intensity ratio (PIR) comparisons for the PNT2-C2 and LNCaP (all spectra), and the sub-stratified clusters: A (PNT2-C2), B (LNCaP—protein dominant) and C (LNCaP—lipid dominant).

PIR	PNT2-$\hbox {C2}_{{All}}$	PNT2-$\hbox {C2}_{{A}}$	$\hbox {LNCaP}_{{All}}$	$\hbox {LNCaP}_B$	$\hbox {LNCaP}_C$
2852 / 2933 ($\hbox {CH}_2$ / $\hbox {CH}_2$ &$\hbox {CH}_3$)	0.30 ± 0.02	0.33 ± 0.02	0.47 ± 0.03	0.09 ± 0.01	0.70 ± 0.03
3015 / 2852 ($=$CH / $\hbox {CH}_2$)	0.19 ± 0.01	0.18 ± 0.01	0.21 ± 0.01	0.30 ± 0.02	0.205 ± 0.004
3015 / 2966 ($=$CH / $\hbox {CH}_{{3}}$,Ch. &CE)	0.065 ± 0.001	0.072 ± 0.002	0.100 ± 0.002	0.041± 0.001	0.129 ± 0.002

Errors are from the propagated SEs.

The profile for Cluster C indicates a greater proportion of lipid-rich signatures relative to the full disease state with 2852 cm^−1^ $\uparrow$ (total lipids), 2894 cm^−1^ $\uparrow$ (lipid-predominant CH, $\hbox {CH}_2$ and $\hbox {CH}_3$ markers), and 3015 cm^−1^ $\uparrow$ (unsaturated lipids) (Fig. 2f). A relative decrease in cholesterol and cholesterol-ester signatures relating to the 2966 cm^−1^ marker in Cluster C shows this subset (56% in Table 2) has even less cholesterol/cholesterol ester. Although Cluster B has a relatively modest weighting in the disease state at 14% within the full, statistically-converged LNCaP dataset (Table 2), it is shown to have a pronounced defining effect on the disease-state phenotype having an up-regulation of protein-dominant signatures at 2933 cm^−1^ $\uparrow$ ($\hbox {CH}_3$) and 2966 cm^−1^ $\uparrow$ (attributed also to the $\hbox {CH}_3$-related component). The designation of LNCaP components that are lipid-rich (Cluster C) or protein-rich (Cluster B) can be further substantiated from peak-intensity ratio analyses (Table 3). The marked down-regulation of the 2852/2933 PIR in Cluster B (0.09) indicates a higher relative proportion of $\hbox {CH}_3$ in this cluster (protein dominant), whereas this marker is markedly up-regulated in Cluster C (0.70) demonstrating more relative $\hbox {CH}_2$ total lipids. The 3015/2852 PIR indicates an up-regulation of unsaturated fatty acids in LNCaP cluster B against the total fatty-acid content relative to the other clusters. However, this PIR in Cluster C (0.205) is effectively equivalent to the value in LNCaP over all spectra (0.21). With these factors considered, the increase in Cluster C of the 3015/2966 PIR (ratio of unsaturated lipids to cholesterol/cholesterol ester) would therefore be predominantly related to a relative decrease in cholesterol and cholesterol-ester components. Supporting literature, which has used Raman spectroscopy to probe various spatially-resolved cell components albeit in the fixed state (i.e., lipid droplets, nucleoli, cytoplasm, etc.), also confirm that the relative increases in signatures for wavenumbers (${\tilde{\nu }}$) $\le$ 2900 cm^−1^ and at ~  3015 cm^−1^ are related to lipid-rich components (such as lipid droplets), and those with relative increases in signatures 2900 cm^−1^ $\le$ ${\tilde{\nu }}$ $\le$ 3000 cm^−1^ are related to protein-rich components (such as nucleoli)^24,43^.

## Discussion

The discovery by unsupervised machine learning in this work of sub-stratified lipid-rich and protein-rich components having defining and distinct disease-related characteristics has implications in the mechanistic understanding of prostate-cancer disease. Increased fatty acid content in prostate-cancer cells, and storage of this within lipid droplets, are defining characteristics of the disease state^39,40^. Prostate cancer cells have significantly higher energy demands. They have adapted to this by using glycolysis to meet these demands either via synthesis of stored cholesterol ester as well as using stored lipids to best repair oxidative damage caused by increased energy usage^44,45^. This work shows LNCaP is more lipid-rich than PNT2-C2, and has a predominant lipid-rich sub-component, which defines its disease state (Cluster C). We believe the predominance of this sub-cluster is directly linked to the LNCaP cell line containing lipid droplets that are greater in size and abundance compared to normal prostate cell lines^46^. Although storage of cholesterol ester within lipid droplets is integral to the disease state, LNCaP has reduced cholesterol-ester amount against other prostate-cancer cell lines, also relative to its own total fatty-acid content and in free-cell cholesterol content as determined in this work (see also supporting references^24,41^). Sorvena et al. has also demonstrated down-regulation of cholesterol and cholesterol ester in LNCaP with up-regulation of other lipids against the normal prostate cell line PNT1 (in particular unsaturated fatty acids) using mass spectrometry^46^. The composition of these droplets has been assessed as being primarily unsaturated fatty acid (e.g., oleic acid via Raman^47^) with a greater ratio of unsaturated fatty acid to cholesterol/cholesterol ester being also determined (this work, also Hislop et al.^24^).

There is a greater amount of lipids in LNCaP compared to the normal cell line as shown in this work, and in other works (e.g.,^46,48^). Prostate cancer cells have a high dependency on lipids for the production of cellular energy, membrane integrity and repair, hormone production, intracellular signalling as well as in other key mechanistic processes^49,50^. The increased availability of lipids via uptake and synthesis, and their accumulation in lipid droplets in prostate cancer, not only preserves lipid homoeostasis and prevents lipids from oxidative damage, it also provides a means of ATP and NADPH production during conditions of metabolic stress (i.e., via mitrochondrial beta-oxidation^49^), when glycolysis-ATP production may be challenged^39^. In this respect, LNCaP is less reliant on glycolysis and more dependent on mitochondrial metabolism, which principally acts to generate its ATP^51^—it has lower levels of glucose consumption and lactate production than other prostate cell lines^52^. LNCaP also has a higher dependency on direct cholesterol use from de novo and extracellular sources^44,53^, with this being linked to reduced cholesterol-ester synthesis. Mechanistically, the cholesterol ester amount in lipid-droplet storage in prostate-cancer cells has been positively linked to the PTEN/PI3K-AKT pathway, the up regulation of which is vital for prostate-cell proliferation and growth^41^. However, PTEN is not expressed by LNCaP^54^ and cholesterol ester is down; therefore, rerouting to induce AKT1 activation to support tumour growth occurs by other means in LNCaP cells, namely via direct cholesterol use^55^. Cholesterol use is an essential component in supporting LNCaP proliferation and maintaining cell-membrane integrity^44^. The unsupervised discovery of a sub-cluster in LNCaP in this work—integral to defining the disease state and having signatures of increased total lipids, reduced cholesterol and cholesterol ester—is therefore a key finding supported and explained by these mechanistic lines of evidence.

The Raman signature and biomarker profile from Cluster B in the LNCaP state indicate a distinct contribution from a protein-rich component within the cell nuclei, which is also a defining feature of the disease state. The highest concentrated component of proteins in the nucleus is the nucleolus^56^. Koh et al. showed multiple nucleoi in LNCaP cells with ~ 80% containing more than one nucleolus^57^. In spatially-resolved measurements on fixed LNCaP cells, Hislop et al. identified intra-nucleus rich regions attributed to nucleoli with higher relative $\hbox {CH}_3$ to $\hbox {CH}_2$ via Raman spectroscopy^24^. The dysregulation of ribosomal proteins in nucleoli are implicated in the pathogenesis of prostate cancer^58^ as is its overexpression, also evidenced specifically in LNCaP^59^. The ribosomal pattern transcript correlates to the type of cancer, and in defining the disease state against normal tissue^60^. Biogenesis implicated by ribosomal differences arising from molecular-scale heterogeneity have been linked to metastatic potential and treatment resistance^61^. Although the precise mechanistic implications of these changes in cancer remain to be fully elucidated, there may be links with the lipogenesis pathway; for example, lack of PTEN results in the inability to suspend proliferation in the event of ribosome overproduction^62^. In the case of LNCaP, this work may therefore be an exemplar where unsupervised machine learning has uncovered intracellular disease-state functions resulting in a lipid-rich component (sub-cluster C) and a protein-rich component (sub-cluster B) which may be synergistically and mechanistically linked.

## Conclusion

We believe this to be the first time that an unsupervised self-organising map approach has been used to classify prostate-cancer at the single-cell level using live-cell Raman spectroscopy data. Using this approach, we have discovered two sub-stratified cancer disease-states in the metastatic LNCaP cell line relative to the normal prostate cell-line, PNT2-C2—a lipid-rich component commensurate with increased lipid-droplets, lipid storage and lipid use in LNCaP and a protein-rich component, which best relates to increased ribosome-density nucleoli. We have proposed that the SOM-classified sub-clusters that define the LNCaP disease state at the single-cell level are mechanistically linked for onward testing. The work demonstrates the unsupervised sub-classification by machine learning of intracellular features that define the disease state in live, intact cells with statistically-robust cell numbers, versus previous efforts that derive biomarkers from the statistical average only, use Raman spatial mapping over small cell numbers and in fixed cells. The method has application in the quantitative discovery of sub-stratified states in prostate cancer, which could better inform patient-specific diagnosis, prognosis and treatment decisions for this highly heterogeneous disease. The method could also inform the discovery of mechanistically-linked features in the disease state, an important application of which could be the design of more effective “broad-spectrum” treatments that can simultaneously target linked disease-state processes.

## Methods

All methods were carried out in accordance with relevant guidelines and regulations under approval from the University of York’s Biology Ethics Committee (https://www.york.ac.uk/biology/current-students-staff/ethics/bec/) (biol-ethics@york.ac.uk). The cell lines used in this study are immortalised cell lines and not primary cell cultures.

### Cell lines used in this study

#### LNCaP

Metastatic prostate cancer cell line of lymph-node origin (left supraclavicular lymph node) isolated by needle aspiration biopsy from a 50-year old male. Luminal-like. Exhibits AR expression (i.e., it is androgen dependent). Prostate specific markers, AR (androgen receptor protein marker), PSA (prostate specific antigen) and PAP (prostatic acid phosphatase), detected. Cell line obtained from ATCC. See Refs.^26,27,30^.

#### PNT2-C2

A well-differentiated, normal prostate epithelial cell line derived from prostate luminal secretory cells obtained from prostate tissue from a 33-year old male post mortem. The cell line was immortalised via transfection with simian virus 40 (SV40). Prostate specific markers, AR (androgen receptor protein marker), PSA (prostate specific antigen) and PAP (prostatic acid phosphatase), not detected. PNT2-C2 is a sub-clone developed from a parental clonal cell line (PNT). Its original purpose was as a means of establishing a consistent in vivo cell-line model of prostate disease states by using it to develop other sub-clonal lines via transfection. Cell line obtained from ECACC. See Refs.^28–30^.

#### Cell culturing

PNT2-C2 and LNCaP cell lines were cultured in T75 tissue culture-treated flasks. They were grown in RPMI (Roswell Park Memorial Institute-1640, Gibco) medium with 10% foetal calf serum (FCS) (R10 media) and 2 mM L-glutamine. The cells were incubated at $37~^{\circ }$C in a humidified atmosphere containing 5% $\hbox {CO}_{{2}}$. No antibiotics were used during standard culture conditions.

#### Sample preparation for Raman spectroscopy

Preparation of the cell samples for Raman spectroscopy analysis follows a three-day protocol. On day one, cells were prepared by washing in PBS followed by incubation in trypsin and re-suspension in R10 media to inactivate the trypsin. The cells were then centrifuged, resuspended in the appropriate media (as above) and counted using a haemocytometer. A $\hbox {CaF}_2$ Raman grade 13 mm (D) × 1 mm (T) disc (Crystran Limited, Poole U.K.) was placed in a 35 mm tissue culture dish. 50,000 cells were plated onto the disc in 200 μl of media and left for 10 min to adhere. Media was then added to a total volume of 2.5 ml, with antibiotic-antimycotic (ABM) solution (Thermo Fisher Scientific) since the discs are not sterile. The dishes were then placed in a $37\,^{\circ }$C incubator. On the second day, media was changed to starvation media (as follows) to synchronise the cells so that they were not in the process of dividing, which would skew results. Starvation media comprised RPMI only, minus FCS and L-glutamine. On the third day for live-cell analysis, the cells were washed three times in HBSS buffer and then 2.5 ml of fresh HBSS buffer was added to the dish. The dish containing the disc (cell sample) was then immediately taken to the Raman microscope for analysis.

#### Raman spectroscopy measurements

Raman point spectra were collected using an HORIBA XploRA micro-Raman with in confocal setting (100 μm pinhole), with 200 μm slit, 532 nm laser wavelength at 3.5 mW laser-power and 2400 lines/mm diffraction grating. A Zeiss Wplan Apochromat 63X (NA = 1.0) Ph3 dipping lens was used. The diffraction-limited, spatial resolution was ~ 1 μm with ± 3 cm^−1^ spectral resolution. Single spectra were collected from the nucleus of randomly selected cells across the population with one spectrum per cell nucleus to ensure minimum laser-dose exposure. Each measurement session per cell sample was no longer than four hours, with up to five cell samples measured per cell line to obtain the statistically-converged spectral numbers. We have shown these measurement parameters and conditions to be non-destructive to live cells with cell viability remaining fully intact via trypan-blue assay testing^63,64^. The cells were also monitored during real-time acquisition to ensure no spectral or optical changes occurred after each Raman measurement.

#### Raman datasets

The Raman spectra were minimally preprocessed using standard methods of baseline subtraction, total-area normalisation, and interpolation (see Ref.^18^ and methods detailed therein). Specifically, the spectra were first cut to the high-wavenumber (2700–3100 cm^−1^) range. The spectral cut, and follow-on baseline subtraction, total-area normalisation, and spectral smoothing with 0.65 cubic spline were performed using the Raman tool set software, version 2.1.0^65^. Due to the HBSS background in the high-wavenumber region of the live-cell spectra, an n = 3 polynomial background subtraction was required. The spectra had minimal background removal using the Raman Tool Set as the inclusion of spectral background has been shown to be beneficial in discriminating cell phenotypes (see for example, Ref.^66^). Each spectrum was also interpolated using code written in IGOR Pro Version 9.01 (WaveMetrics, Inc., Lake Oswego, OR, USA) to ensure the same wavenumber increments across the spectra for follow-on PCA analyses. Convergence of the average spectrum, twice the standard deviation (2xSD) and standard-error of the mean (SE) for increasing numbers of spectra ensured the data sets were statistically-representative of population-level, live-cell and dried-cell states. Statistical convergence is shown for LNCaP with 130 live-cell spectra and PNT2-C2 with 154 live-cell spectra collected in the high-wavenumber region (cf. Supplementary Fig. S1). Strict convergence of the statistical quantities ensured experimental variability and molecular-scale heterogeneity were fully accounted for.

#### Peak intensity and peak-intensity ratio (PIR) analyses

Gaussian peak-fitting was performed on the statistically-converged, normalised average spectra per cell line across linear-baselined, local spectral windows using the Multipeak Fitting 2 function in IGOR Pro Version 9.01 (WaveMetrics, Inc., Lake Oswego, OR, USA). The fitted peak-intensities were used to obtain disease-state biomarkers that differentiate the live and dried disease-states via peak-intensity subtractions. Robust biomarkers were determined under criteria where the magnitude of the disease-state marker needed to be significantly greater than the combined, fitted standard-error uncertainties corresponding to each de-convolved band per individual spectrum. Peak-intensity ratios between key bands were also calculated with the uncertainty of the PIR obtained from the propagated sum of the relative standard-error uncertainties of the composite bands.

#### Principal component and linear discriminant analyses

Principal component analysis (PCA) was performed with loadings and scatter plots produced using code written in R and executed in RStudio version $2022.07.2+576$^67^. After PCA, linear discriminant analysis (LDA) was performed followed by leave-one-out cross-validation using code written in R and executed in RStudio version $2022.07.2+576$^67^. The optimal number of PCs for LDA inclusion was determined by assessing the stability of the leave-one-out cross validation result as a function of the number of PCs included about the Kaiser criterion point with respect to the proportional variance captured per principal component (PC), and cumulative variance captured as a function of increasing principal components (PCs). Leave-one-out cross validation (LOOV) was used to determine the classification accuracy, as well as to check the numerical stability of the PCA-LDA result. Histograms for the LDA classification result were then generated. An LDA-weighted, loadings summation over the included PCs (sum-weighted loadings) was obtained, as well as measures for the group-mean separations, within-group variance, and ratio of the within-to-between-group variances determined using in-house code written in R. Biomarkers from the loadings results were deemed viable if they were significantly prominent above the fluctuating loadings background.

### SOM method

(i) *MiniSom and MySom codes:* MiniSom^68^ is an open source Python package used for the computational SOM analysis. The Python module MySom was developed as a subclass of MiniSom, which contains methods for normalisation of Raman spectral data and plotting SOM outputs^69^. The source code for MySom is available at github.com/thenakedcellist/prostate. The SOM is built using multiple input parameters: the map network comprising the *x* and *y* dimensions and number of *n* nodes, the starting neighbourhood radius, $\sigma$(0), the starting learning rate, $\alpha$(0), and the number of iteration steps in the learning process.
(ii) *Raman input datasets:* The dataset for SOM analysis contains spectral data from an unknown (blinded) number of PNT2-C2 (normal prostate) and LNCaP (prostate cancer) cell lines, total 284 observations. The unlabelled dataset is stored as two files, the first containing a one-dimensional array of length 1056, each column containing a value for the wavenumber (cm^−1^). The second file contains a two-dimensional array containing measured arbitrary intensity values that correspond to each wavenumber. The data for the samples therefore form a 284 $\times$ 1056 array.
(iii) *SOM algorithm:* The following SOM algorithm is implemented using MiniSom and MySom. Its parameterisation is in sub-section (iv), which follows.

1. A rectangular SOM of *x* and *y* dimensions is defined, which has $x \times y = n$ nodes.
2. A normalised weight array $w_k=[w_{k1},...,w_{kN}]$ is created for each of the *k* SOM nodes. Here, $w_{kj}$, are the weight elements, which are initialised with random values chosen from 0 to 1, and *j* is the element number, where $j=\{1,N=1086\}$ are the wavenumber measurements. Each spectrum $i=1$ to 284 in the total data set is also assigned to an array $v_{i}=[v_{i1},...,v_{iN}]$, which is normalised by its Frobenius norm. The iteration counter *t* is initialised at zero and runs to $t=t_{max}$.
3. An input vector $v_{i}$ is randomly chosen from the data set, with the Euclidean distance calculated between it and the weight array $w_k=[w_{k1},...,w_{kN}]$ for each node *k* in the SOM, such that $D_{v_{i}w_k}=\sqrt{\sum _{j=1}^{N}(v_{ij}-w_{kj})^{2}}.$ The node *k* with the closest Euclidean distance to the input vector is then defined as the “best-matching unit” (BMU).
4. The weight arrays corresponding to the nodes are modified in the next $t+1$ iteration using the function, $w_k(t+1) = w_k(t)+\alpha (t)h_{k}(t)(v_i-w_k(t))$. Decay functions may be any function of *t* that allows $\sigma (t)$ and $\alpha (t)$ to decrease with increasing *t*^70^. Here, $\alpha (t)=\frac{\alpha (t-1)}{a+\frac{t}{0.5t_{max}}}$ is the learning rate, which asymptotically decays, and $h_{k}(t) = e^{-d^2/2\sigma (t)^2}$ is the neighbourhood function, which determines the size of the “neighbourhood” radius around each BMU. Here, *d* is the Euclidean distance from the neighbouring nodes to the BMU and $\sigma (t) = \frac{\sigma (0)}{1+t*C}$ where $C = \frac{(\sigma (0)-1)}{t_{max}}$. The nature of the decay functions for $\alpha (t)$ and $\sigma (t)$ allow for early coarse organisation and later fine organisation of the data^70^. That is, with each step, the neighbourhood radius and learning rate decay monotonically to achieve gross clustering of widely-spaced nodes early on in training, and fine tuning of clusters during later iteration steps by only acting on close neighbours.
5. Steps 4 and 5 are iterated through until $t=t_{max}$ is reached.

(iv) *Parameterisation:* The parameters for the SOM are not known *a priori*, rather Kohonen stated they could be determined via trial and error and visual inspection of the results, depending on the granularity of data clustering desired^70^. We used a two-dimensional lattice and rectangular topology, which is easier to interpret compared to other SOM representations (e.g., hexagonal geometry). As guidance and to aid computational efficiency, most sources recommend using a map size of 5$\sqrt{n}$ nodes, where *n* is the number of observations, following early work by Vesanto^71^. Kohonen also suggested that the *x* and *y* dimensions of the map could be configured using the ratio of the two highest eigenvalues of the autocorrelation matrix for the input data^70^. To ensure robust clustering with a rectangular lattice, the *x* and *y* dimensions should also not be the same; breaking symmetry of the map in this manner ensures faster learning^72^. In applying these recommendations, our initial testing resulted in an elongated SOM of dimensions 23 $\times$ 6^69^ (see also Fig. S2(a) in the Supplementary). Further testing led to a map size of 10 $\times$ 14 = 140 nodes (this work) allowing for a greater map-area to side-length ratio given the open boundary conditions, and better visualisation of the results overall. Importantly, the change between the two geometries did not affect the outcome of the SOM findings (cf. Figs. S2(c), (d) and (e) in the Supplementary), showing robustness in the unsupervised classification result against the optimised parameter set as next described. 

The SOM training used 10$\times 10^{5}$ iteration steps to achieve convergence, which corresponds to the value Kohonen used in his original SOM simulations^32^, with optimum parameters determined as $\sigma (0)=3.0$, $\alpha (0)$ = 0.75 and random seed = 1^69^. A threshold distance for cluster inclusion was determined as 0.72 corresponding to the SOM heat-map scale (see the scale bar on the right-hand side of the SOM in Fig. 2a). This value corresponds to the distance value whereby clefts between clusters (cluster borders) occur. The distance between neighbouring nodes is artificially low at the map edges and corners, as only three edge and two corner neighbours are used in weight calculations within the MiniSom code. This led to a perceived increase in nodal density as the code does not allow wraparound of the map in the data space. This aspect was accounted for in our method by not allowing observations mapping to edge and corner nodes to define borders between clusters (due to artificial increased density/clustering). Observations that map to edge nodes are still included for analysis if they have a nearest-neighbour internal node, which defines a cluster. Corner nodes, which have nearest-neighbour nodes that are edge-nodes only, are fully excluded from cluster definitions in the SOM.

## Supplementary Information

Supplementary Information.

## Data Availability

The datasets generated and/or analysed during this study are available by request from Y.H.
