# Supplementary material for: Unsupervised self-organising map classification of Raman spectra from prostate cell lines uncovers substratified prostate cancer disease states
Source: Sci Rep. 2025 Jan 4;15:773. doi: 10.1038/s41598-024-83708-6 (PMC11700215; doi:10.1038/s41598-024-83708-6)
Supplement: Supplementary file 1 — Supplementary Information. [file 41598_2024_83708_MOESM1_ESM.pdf]

Supplementary Information

Unsupervised self-organising map classification of Raman spectra from prostate cell lines uncovers substratified prostate cancer disease states

Daniel West,[1] Susan Stepney,[1] and Y. Hancock*[2,3]

[1]*Department of Computer Science, University of York, Heslington, York, YO10 5GH, U.K.*

[2]*School of Physics, Engineering and Technology,*

University of York, Heslington, York, U.K., YO10 5DD

[3]*York Biomedical Research Institute, University of York, Heslington, York, U.K., YO10 5DD**

* y.hancock@york.ac.uk

A. Statistical convergence

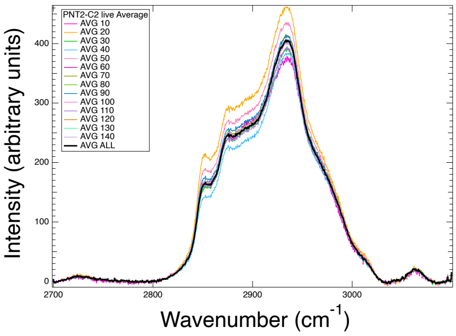

(a)

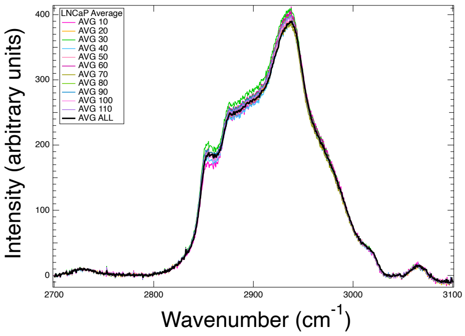

(b)

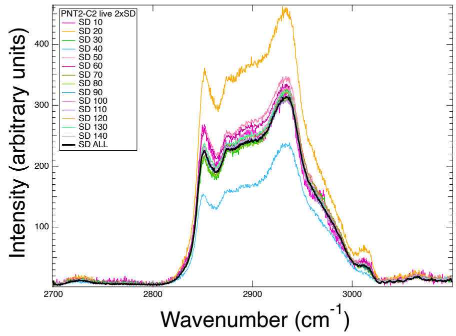

(c)

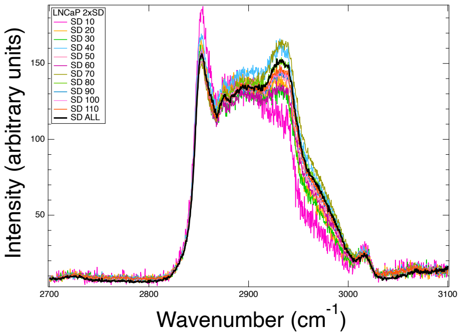

(d)

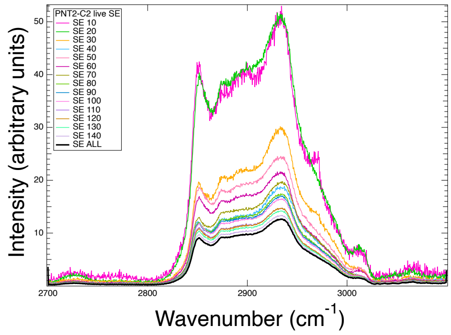

(e)

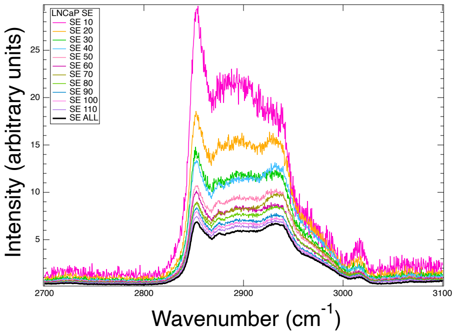

(f)

FIG. S1. Statistical convergence of the high-wavenumber spectra as a function of increasing numbers of spectra in the spectral average for the (a) PNT2-C2 average, (b) LNCaP average, (c) PNT2-C2 twice the standard deviation, (d) LNCaP twice the standard deviation, (e) PNT2-C2 standard error and (f) LNCaP standard error.

B. SOM reproducibility assessment

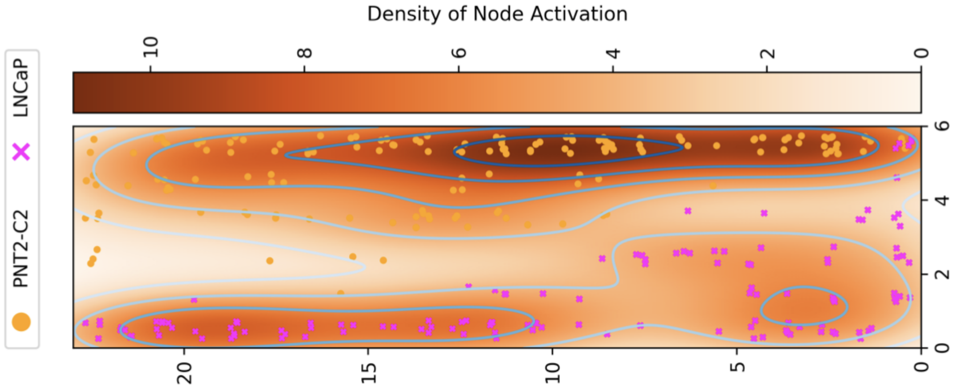

(a)

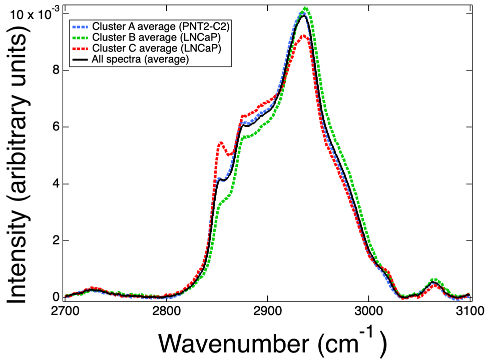

(b)

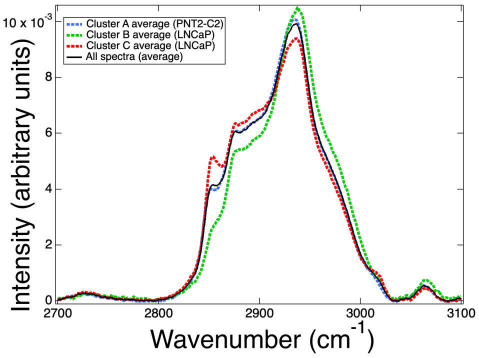

(c)

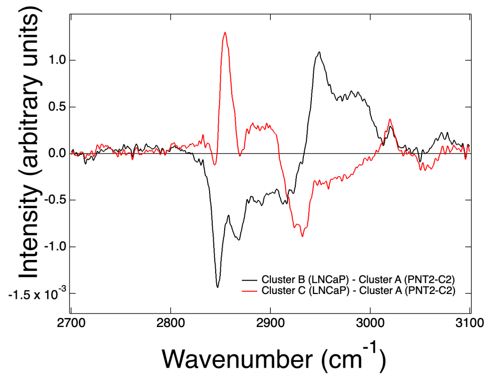

(d)

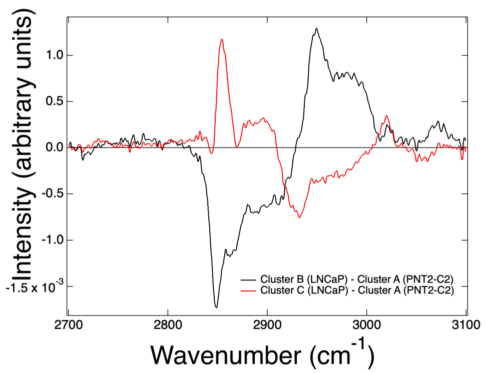

(e)

FIG. S2. (a) A 23 $\times$ 6 unsupervised SOM was originally tested showing the three sub-clusters defined [67]. Results in (b) and (d) from the 23 $\times$ 6 SOM show excellent similarity to those obtained in this work in (c) and (e) for the 10 $\times$ 14 SOM (cf. Fig. 2 in the main text), thereby demonstrating robustness of the parameters and the unsupervised classification findings.

C. SOM statistical significance

PCA point of interest (spectrum)	SOM node	SOM-assigned **distance threshold for inclusion = 0.72**	SOM unsupervised classification	SOM distance score (DS)
L1	(10,3)	**Included**, but dist. (0.71) i.e., borderline for inclusion	Cluster C LNCaP	0.71
L2	(8,0)	**Excluded edge node**. Has empty internal neighbour node (8,1).	Non assigned	0.33
L3	(9,0)	**Included edge node**. Has filled internal neighbour node (9,1)	Cluster C LNCaP	0.33
L4	(13,9)	**Excluded corner node**; mixed	Non assigned	0.31
N1	(0,6)	**Excluded edge node.** Has empty internal neighbour node (1,6)	Non assigned	0.21
N2	(5,6)	**Included**. Has filled internal-cluster neighbour node (5,7) and empty between-cluster neighbour node (5,5)	Cluster A PNT2-C2	0.64
N3	(0,8)	**Excluded edge node.** Has empty internal neighbour node (1,8)	Non assigned	0.17
N4	(8,5)	**Included.** Has filled internal neighbour node (8,6) and empty between-cluster neighbour node (8,4)	Cluster A PNT2-C2	0.63

TABLE S1. Mapping of the SOM nodes [Figure 2(a) main article] and PCA points of interest [Figure 2(b) main article]. The unclassified SOM inclusion or exclusion decision, and distance score relative to the distance threshold score (=0.72) are also shown.

From the above table we note:

1. Where there has been a SOM assignment, the unsupervised SOM has made the correct classification (LNCaP => Cluster C and PNT2-C2 => Cluster A).

2. The SOM-classified PCA points of interest have very small weighting in the A and C cluster populations (cf. Table 2 in the main article). Two spectra in the PCA points of interest have been assigned to Cluster A (N2 and N4) ~2% of the 121 total spectra in the SOM-classified sub-cluster. Two spectra have been assigned to Cluster C (L1 and L3) ~3% of the 73 total spectra in the SOM-classified sub-cluster.

3. None of the PCA points of interest map to LNCaP Cluster B, demonstrating the significance of this SOM-classified sub-cluster.

4. L1, which is a PCA outlier, has borderline SOM inclusion.

We inspected L1 in more depth due to it being a PCA outlier with a SOM distance score of 0.71 making it borderline for inclusion in the SOM classification. L1 is from the (10,3) SOM node. As can be seen in Figure S3, L1 (spectrum LNCaP) is a PCA outlier likely because of its (1) spectral noise and (2) an accidental issue with baselining from 3050 to 3100 cm-1 (no other spectra had this issue). Against the cluster means, the unsupervised SOM has correctly assigned the L1 LNCaP spectrum to be most closely mapped to LNCaP (Cluster C). In addition, the SOM recognises L1

as being borderline for classification (distance score 0.71 relative to the 0.72 cutoff). Its inclusion in Cluster C is only 1/72 = 1% weighting.

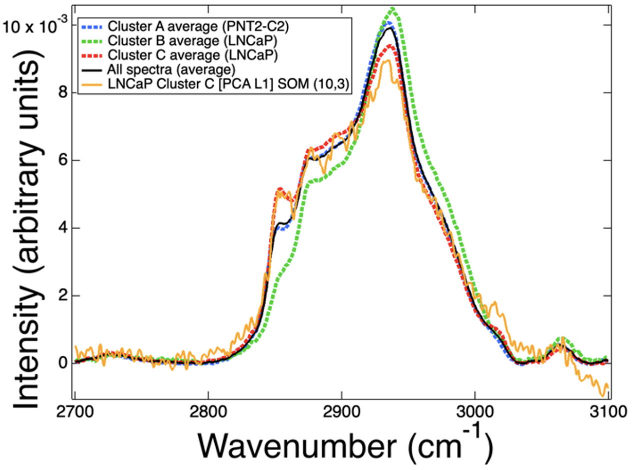

(a)

FIG. S3. Spectrum from the SOM borderline case (10,3) (TD = 0.71), which mapped to the L1 PCA outlier versus the spectral average for each unsupervised SOM-classified cluster.

Therefore, based on our assessment of the PCA points of interest, we conclude that PCA has affirmed the statistical significance of the unsupervised SOM classification and sub-clustering.
